# Novel insights into tomato leaf curl New Delhi virus introduction and evolution in Southeastern France using an advanced long-read sequencing workflow

**DOI:** 10.1371/journal.ppat.1014463

**Published:** 2026-08-07

**Authors:** Atiwich Patthamapornsirikul, Victor Golyaev, Eric Verdin, Claire Goillon, Hindi Boolell, Sam Dierickx, Catherine Wipf-Scheibel, Grégory Girardot, Wim Dumon, Sara Cleemput, Koen Deforche, Hervé Vanderschuren, Cécile Desbiez

**Affiliations:** 1 INRAE, Pathologie Végétale, Montfavet, France; 2 Department of Biosystems, Tropical Crop Improvement Laboratory, Crop Biotechnics, KU Leuven, Leuven, Belgium; 3 KU Leuven Plant Institute (LPI), KU Leuven, Leuven, Belgium; 4 Association Provençale de Recherche & d’Expérimentation Légumière – APREL, Saint-Rémy-de-Provence, France; 5 Emweb BV, Herent, Belgium; 6 Plant Genetics and Rhizospheric Processes Laboratory, Gembloux Agro BioTech, University of Liège, Gembloux, Belgium; Tel Aviv University, ISRAEL

## Abstract

The Mediterranean population of tomato leaf curl New Delhi virus (ToLCNDV-ES) is characterized by a high genetic uniformity, distinguishing it from its Asian counterparts. ToLCNDV-ES is thought to have a monophyletic origin, likely resulting from a single recombination event, prior to its spread throughout the Mediterranean region. Following its first detection in southeastern France in 2020, ToLCNDV-ES re-emerged in France in 2022. Our analysis based on advanced long-read sequencing, circular DNA profiling, and phylogeny indicates both local persistence of French ToLCNDV-ES and multiple independent introduction events. Signatures of positive selection were identified in French ToLCNDV-ES populations, whereas no clear evidence of recombination was found. Bayesian time-structured phylogenetic analyses suggest that introductions in France occurred between 2018 and 2021 from the major ToLCNDV-ES clade, while several Italian ToLCNDV-ES isolates diverged prior to the virus introduction in the Mediterranean basin. Overall, this study demonstrates the value of an optimized long-read sequencing approach for resolving circular DNA virus diversity, and sheds light on the complex evolutionary history of ToLCNDV-ES in the Mediterranean Basin, particularly in southeastern France.

## Author summary

Understanding how emerging plant viruses invade new regions and evolve after introduction is a central challenge in pathogen biology. Here, we investigate the emergence and spread of the Mediterranean strain of tomato leaf curl New Delhi virus (ToLCNDV-ES) in southeastern France following its first detection in 2020 and subsequent re-emergence in 2022. While ToLCNDV-ES populations across the Mediterranean basin have previously been reported to exhibit remarkable genetic uniformity, the processes underlying their persistence and dissemination remain poorly understood. Using an advanced long-read sequencing workflow combined with circular DNA profiling and phylogenetic analyses, we characterize the evolutionary dynamics of ToLCNDV-ES populations at unprecedented resolution. Our analyses reveal evidence for both local persistence of viral populations and multiple independent introduction events into France, highlighting the complex invasion dynamics of this emerging begomovirus. We detect signatures of positive selection in French ToLCNDV-ES populations. Our analyses suggest that introductions into France likely occurred between 2018 and 2021 from the major Mediterranean ToLCNDV-ES lineage. Moreover, our study demonstrates the value of an advanced long-read sequencing strategy for studying circular DNA viruses, building upon a framework previously described. By enabling accurate reconstruction of full viral genomes and population diversity, this approach provides a powerful tool to investigate the emergence, spread, and evolution of plant viruses. We believe that this methodological advance, combined with our analysis of ToLCNDV-ES introduction dynamics, offers insights that are broadly relevant for understanding the evolutionary trajectories of emerging viral pathogens.

## 1. Introduction

Southern France represents an important agricultural hub for vegetable and horticultural crop production, particularly cucurbit crops such as courgette, melon, and cucumber, which are cultivated in either open fields or greenhouses. This economically important sector is regularly threatened by a wide range of plant viruses, predominantly aphid-transmitted species [1–3]. These endemic viral agents are not only widespread but also exhibit complex molecular population structures characterized by high mutation rates, frequent genetic recombination, and repeated introductions leading to the emergence of novel variants [1–7]. In addition to the endemic aphid-transmitted viruses, tomato leaf curl New Delhi virus (ToLCNDV) was first reported in southeastern France in 2020 [8] and has since become more frequent in the region.

ToLCNDV (*Begomovirus solanumdelhiense*)*,* a bipartite begomovirus transmitted by the whitefly *Bemisia tabaci* [9,10], has emerged over the last decades as one of the most significant plant viruses in Asia and the Mediterranean Basin. First identified in the Indian subcontinent, Asian ToLCNDV isolates display a broad host range, infecting major crops notably within the Solanaceae (tomato, pepper, potato), Malvaceae (cotton and okra), and Cucurbitaceae (zucchini, melon, cucumber, and squash) [9,11,10]. In the Mediterranean Basin, ToLCNDV was first detected in Spain in 2012 and subsequently reported to spread throughout Europe and the wider Mediterranean region [12,9,13,8]. The ToLCNDV strain found in the Mediterranean—commonly referred to as ToLCNDV-ES—has a more restricted host range than Asian isolates, primarily infecting cucurbit species under natural conditions [14,15,10]. ToLCNDV-ES exhibits a relatively homogenous molecular population, distinct from the Asian isolates, and is thought to have arisen through interspecific recombination [14,16]. Its low genetic diversity suggests that the virus was introduced into the Mediterranean region through a single event, followed by rapid spread across the Basin [15,17,18,10]. Despite the overall genetic uniformity, the virus shows evidence of ongoing evolution through the accumulation of mutations and occasional recombination events [17,19].

As in other plant viruses, selection acting on the viral diversity generated by genetic reassortment, mutations, and/or recombination may enhance the adaptability of the virus to diverse environmental conditions and host species, supporting its persistence and spread [5], and potentially enabling it to overcome resistance and evade control strategies [20,21]. For an emerging pathogen such as ToLCNDV, investigating molecular diversity using robust analytical tools is essential for understanding viral ecology, outbreak dynamics and mechanisms of emergence, as well as for developing sustainable management strategies. Advances in molecular techniques, particularly high-throughput sequencing (HTS), have enabled detailed characterization of viral genomes by uncovering fine-scale genetic variations and population structure. HTS-based analyses of viral genomes allow in-depth profiling of inter- and intra-species mixed infections, detection of recombination events, and assessment of within-host diversity, far beyond the capabilities of first-generation sequencing techniques [22,23]. However, accurate genome reconstruction from short-read HTS data can be challenging when viral populations contain multiple closely related variants, potentially leading to artefactual recombinants. This limitation can be overcome by third-generation long-read sequencing approaches. Accordingly, Oxford Nanopore technology (ONT) has been shown to provide cost-effective and high-throughput solutions for accurate reconstruction of virus variants [24]. The major limitation of ONT is its relatively high per-read error rate. However, this can be substantially reduced for circular DNA viruses by performing rolling circle amplification (RCA) prior to sequencing, followed by reconstruction of high-quality consensus genome sequences using established bioinformatic tools [25,24].

To elucidate the evolutionary dynamics of ToLCNDV following its emergence in southern France, we applied a high-throughput sequencing workflow based on ONT sequencing and the online tool Genome Detective Platform [26,24] to perform whole-genome analyses of both ToLCNDV DNA-A and DNA-B components. These analyses allowed us to investigate phylogenetic relationships, infer introduction patterns, identify potential recombination events, and detect signatures of natural selection, thereby providing new insights into the mechanisms shaping the molecular evolution of ToLCNDV.

## 2. Results

### 2.1. Nanopore sequencing accurately reconstructs full-length French ToLCNDV genomes, as confirmed by Sanger sequencing

**Fig 1:**
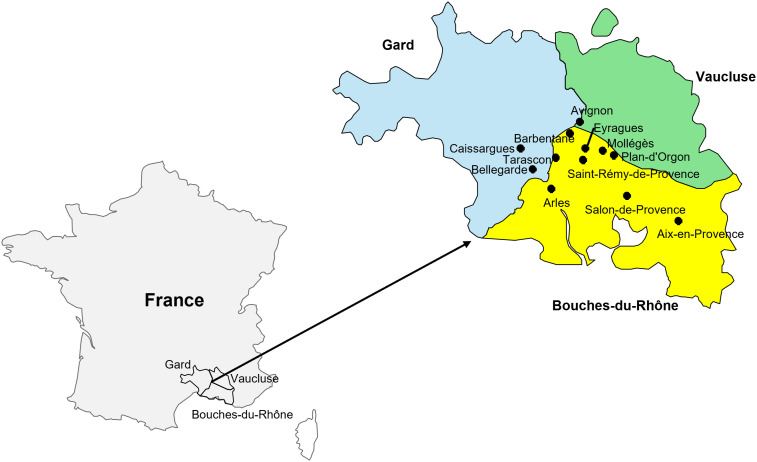
Sampling sites of ToLCNDV in southern France across the départements of Gard (Blue), Vaucluse (Yellow), and Bouches-du-Rhône (Green), covering an approximate area of 1,180 km2. Administrative boundaries are based on Natural Earth country and cultural vector data, with France and subnational boundaries used for spatial reference. Basemap data were obtained from the Natural Earth dataset All spatial analyses and figure generation were conducted in R 4.5.3 using the packages sf, ggplot2, dplyr, ggrepel, rnaturalearth, and rnaturalearthdata. Natural Earth datasets are public domain and freely available for use under the terms described at: https://www.naturalearthdata.com/about/terms-of-use/. Base layer data (country boundaries and cultural vectors) are available here: https://www.naturalearthdata.com/downloads/10m-cultural-vectors/.

ToLCNDV was first identified in autumn 2020 in six infected zucchini plants, sampled at three different sites located in the Bouches-du-Rhône and Gard départements. The virus re-emerged in 2022 at three sites in Bouches-du-Rhône, near the original 2020 outbreak area. An increase in viral detection was observed in 2023 and 2024, during which 41 additional samples were collected within a 60 km radius across the départements of Gard, Bouches-du-Rhône, and Vaucluse (Fig 1). Most infected samples were obtained from zucchini, whereas a limited number were collected from melon, cucumber and the weed species *Ecballium elaterium* and *Datura stramonium* (S1 Table). No ToLCNDV infections were detected in watermelon by ELISA and routine PCR [27,8].

Nanopore sequencing data were generated for 48 French isolates and for the Spanish reference isolate ES13–35 using the pipeline described in [24]. The number of full-length contigs per isolate ranged from 282 to 92,789 (mean = 14,517). The DNA-A/DNA-B ratio among full-length contigs varied from 0.03 to 1.26. For each sample, the most abundant haplotype for each DNA represented, on average, 76% of the reads for DNA-A and 70% of the reads for DNA-B. However, in six samples for DNA-A and 12 samples for DNA-B, the most frequent haplotype accounted for less than 50% of the reads, while one or two additional haplotypes each represented more than 20% of the reads (S1 Fig).

Within each sample, haplotypes could differ by only one or two single nucleotides polymorphisms (SNPs), or by as many as 30 mutations for DNA-A and more than 70 mutations for DNA-B, indicating that they represent different molecular subgroups. The four most abundant haplotypes in each sample together represented 96.3% of the reads for DNA-A and 90% of the reads for DNA-B (S1 Fig).

Samples from the weeds *Datura stramonium* and *Ecballium elaterium* tended to harbor more complex viral populations (S1 Fig), but the number of samples available for each species was too limited to assess whether this represents a general trend associated with long-term infection in these hosts.

Among the major haplotypes in each sample, three isolates for DNA-A and seven isolates for DNA-B contained variants that belonged to molecular subgroups different from that of the major haplotype. One isolate for DNA-A and six isolates for DNA-B were considered to clearly represent multiple infections. In the three remaining cases, the total number of reads obtained for the sample was limited, resulting in only 30–50 reads being assigned to the minor haplotype (S1 Fig). Including negative controls in all the ONT sequencing pools and/or performing technical replicas of the sequences would have allowed to be more accurate in the determination of threshold for very minor variants, even if it had no strong impact on the overall results of the analyses.

For isolate VI231143, low-frequency variants were detected for both DNA-A and DNA-B. For isolate EV231242, the evidence for mixed infection was clear for DNA-B but ambiguous for DNA-A. Thus, in both cases, mixed infections are likely to be genuine for both genomic components, but the sequences of the minor variants were not included in the phylogenetic analyses.

Within each sample, no attempt was made to associate specific DNA-A and DNA-B haplotypes based on their relative frequencies in the ONT sequencing dataset.

The Sanger sequences were 100% identical to the major haplotype obtained by Nanopore sequencing for the reference isolate ES13–35 for both ToLCNDV DNA-A and DNA-B components. Sanger sequences derived from cloned amplicons were either identical to the dominant haplotype identified by Nanopore sequencing of the original uncloned sample or differed by one or two SNPs. Most of these SNPs were unique among the Mediterranean isolates, suggesting that they likely resulted from amplification or cloning artefacts. Partial Sanger sequencing of uncloned amplicons further confirmed identity with the major Nanopore-derived haplotypes or revealed ambiguous positions corresponding to SNPs distinguishing major and minor haplotypes, thereby reflecting the underlying viral population structure within the host plant (S2 Table). Additionally, for the mixed-infected isolates VI231145 (for both DNA components) and VI231242 (only for DNA-B) (S1 Table, S1 Fig), partial Sanger sequencing chromatograms showed multiple peaks at the positions corresponding to the expected SNPs, as well as a frameshift corresponding to the expected indel in DNA-B of VI231242 (S1 Fig).

Genome organization of the French ToLCNDV isolates was consistent with that described for ToLCNDV, comprising seven ORFs in DNA-A and two in DNA-B. Predicted protein lengths were overall conserved relative to other ToLCNDV-ES isolates, with only a few deviations that were validated by Sanger resequencing. For the AC1 (Rep) protein, four isolates (FR416-1, FR240005, VI231143 and VI231240) contained an additional initiation codon at positions 2671–2673, located 90 nt upstream of the canonical AC1 start codon at positions 2581–2583. This feature would potentially result in a 30-amino-acid N-terminal extension. The same upstream ATG was detected in one Spanish isolate (accession MH577727).

Two French isolates originating from the same site (EV240029 and EV240030) contained a premature stop codon within the AC1 coding region, resulting in a Rep protein truncated by nine amino acids at its C-terminal end. In isolate EV240030, a minor haplotype carrying the premature stop codon (EV240030-0-4, accession PX842865) co-occurred with a dominant haplotype lacking the stop codon (EV240030-0-3, accession PX842864) (S2 Table). The biological significance of these AC1 length variations remains unknown.

Within the putative AC5 ORF, whose function remains unknown for ToLCNDV [10], several isolates contained a premature stop codon at position 26. This mutation suggests that a truncated protein of 120 amino acids, initiated from a downstream start codon, may be expressed instead of the full-length 161-amino-acid form.

### 2.2. Limited recombination and evidence of site-specific positive selection in Mediterranean ToLCNDV populations

Using GARD to analyse recombination in the Mediterranean ToLCNDV isolates, a single breakpoint was detected in DNA-B (position 2684, corresponding to Rep cleavage site), although with weak support (Δc-AIC = 290.007 compared with the null model, and Δc-AIC = 774.977 compared with a single-tree multiple-partition model). RDP4 identified a limited number of putative recombination events in both DNA-A and DNA-B. For DNA-A, event 1 involved MF967015.1 (Tunisia) as the major parent and VI240544 (France) as the minor parent (p-values: GENECONV = 0.1202, BootScan = 0.00606, SiScan = 0.0196). For DNA-B, event 1 involved MH577630.1 (Morocco) and MF967022.1 (Tunisia) (p-values: BootScan = 0.02485, SiScan = 0.000595). However, parental assignments were inconsistent across methods, and statistical support remained weak.

Split decomposition analysis using SplitsTree showed no strong evidence of recombination. Phi tests yielded non-significant results for DNA-A (p = 0.066; p = 0.47 for French isolates only) and DNA-B (p = 0.17; p = 0.49 for French isolates) as all p-values were above the 0.05 threshold. In the split decomposition DNA-A network, sequence VI240545 appeared as a potential outlier (S2 Fig). When RDP4 was applied specifically to VI240545 DNA-A together with a subset of 16 French isolates from subgroups A1 and A2, a recombination signal was detected by the MaxChi, Chimaera, Siscan and 3Seq methods (p = 1.9 10^-3^ to p = 1.6 10^-2^). Although VI240545 may therefore represent a recombinant, the limited overall genetic diversity of DNA-A reduces the strength and interpretability of this signal.

**Fig 2:**
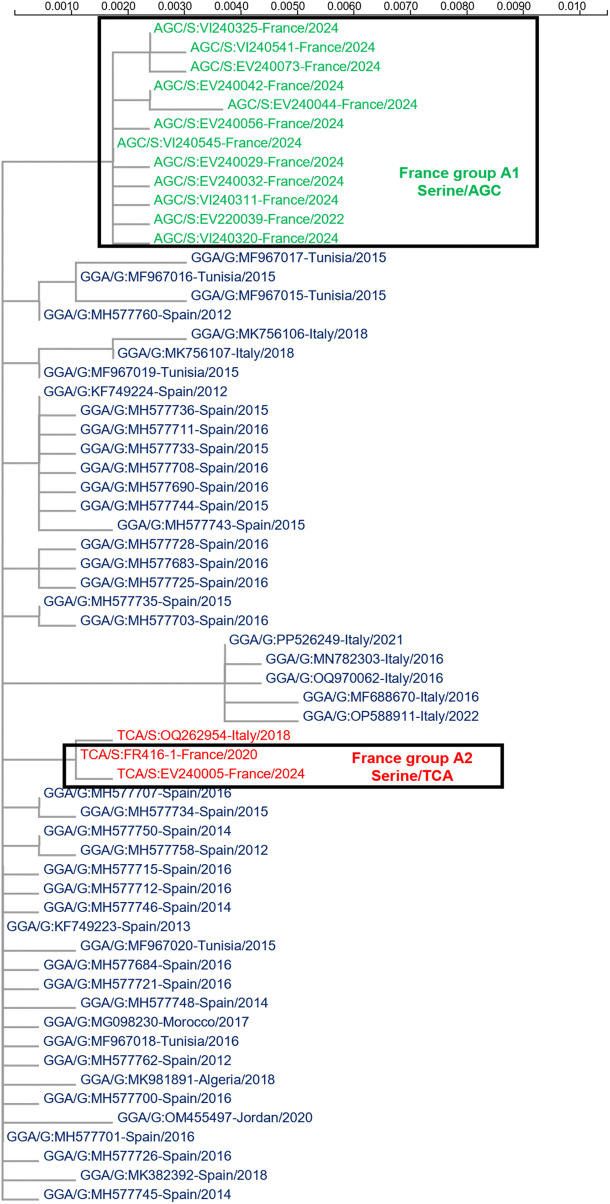
Phylogenetic relationships among nucleotide sequences encoding the coat protein (AV1) of Mediterranean ToLCNDV isolates (non-identical sequences only). The scale bar represents genetic distances inferred under a GTR substitution model. Branch colors indicate selection regimes, with green and orange denoting evidence of positive selection (dN/dS > 1) and blue indicating dN/dS ≤ 1. The color gradient was quantified using Empirical Bayers Factors (EBF) as estimated by the MEME (Mixed Effects Model of Evolution) method (www.datamonkey.org).

FUBAR analysis detected evidence of positive selection at a single site among the 256 codons of the coat protein (CP) in French and Mediterranean ToLCNDV isolates (posterior probability 0.960). This site corresponds to amino acid position 147, where most Mediterranean isolates carried a glycine, whereas all French isolates encoded a serine, specified by either AGC (subgroup A1) or TCA (subgroup A2) (Fig 2). Codon 147 was also identified as positively selected by MEME (p = 0.002). Among non-French Mediterranean isolates, only a single Italian isolate (ZU-Le18, accession OQ262954) encoded a serine at this position. Notably, this mutation also affects the partially overlapping putative AC5 ORF, which is encoded in antisense orientation relative to CP. In subgroup A2 isolates and in the Italian isolate ZU-Le18, a TGA stop codon (corresponding to the TCA in the CP frame) occurs at codon 26 of AC5. This likely results in expression of a truncated 120-amino-acid protein initiated from a downstream start codon at position 42, instead of the full-length 161-amino-acid form, as previously reported for ZU-Le18 [19].

### 2.3. Distinct diversification of DNA-A and DNA-B components reveals reassortment dynamics in French ToLCNDV

**Fig 3:**
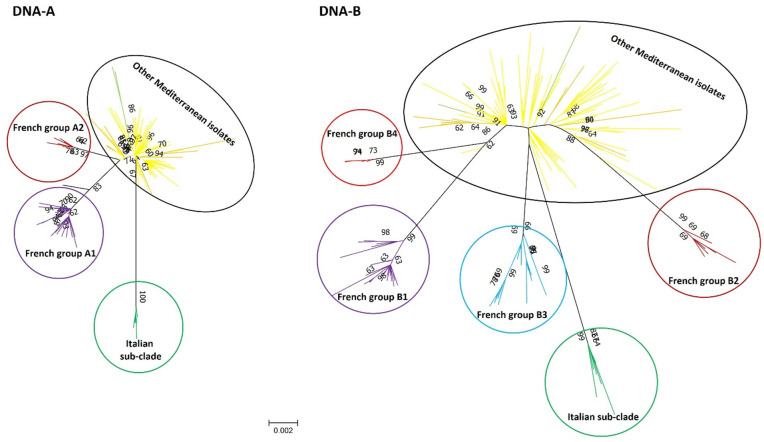
Maximum likelihood trees of Mediterranean Tomato leaf curl New Delhi virus (ToLCNDV) isolates based on full-length DNA-A and DNA-B sequences. The Italian sub-clade is highlighted in green. French lineages are shown in distinct colors, with two subgroups identified for DNA-A (A1 in purple and A2 in dark red) and four subgroups for DNA-B (B1 in purple, B2 in dark red, B3 in light blue, and B4 in red). Bootstrap values (>60%; n = 1,000 bootstraps) are indicated at the corresponding nodes. Spanish isolates are represented by yellow branches, other Italian isolates by light green branches, and isolates from North Africa and Middle East (Algeria, Tunisia, Morocco and Jordan) by orange branches.

Phylogenetic analysis of French ToLCNDV isolates from 2020 to 2024 confirmed that all sequences clustered within the Mediterranean clade for both DNA-A and DNA-B (Fig 3). DNA-A sequences showed limited genetic variation, sharing 98–99% nucleotide identity compared with other Mediterranean isolates. DNA-B exhibited slightly greater divergence, with 97–98% identity relative to Mediterranean ToLCNDV sequences.

Within the French dataset, the two genomic components displayed distinct phylogenetic structuring. DNA-A sequences segregated into two well-supported subgroups, A1 and A2, with low intragroup diversity (0.0026 for A1 and 0.0017 for A2) and moderate intergroup divergence (0.0103). In contrast, DNA-B sequences were more heterogeneous and partitioned into four distinct phylogenetic subgroups (B1-B4). Intragroup diversity ranged from 0.0015 (B4) to 0.0050 (B1), whereas intergroup divergence ranged from 0.020 to 0.024.

**Fig 4:**
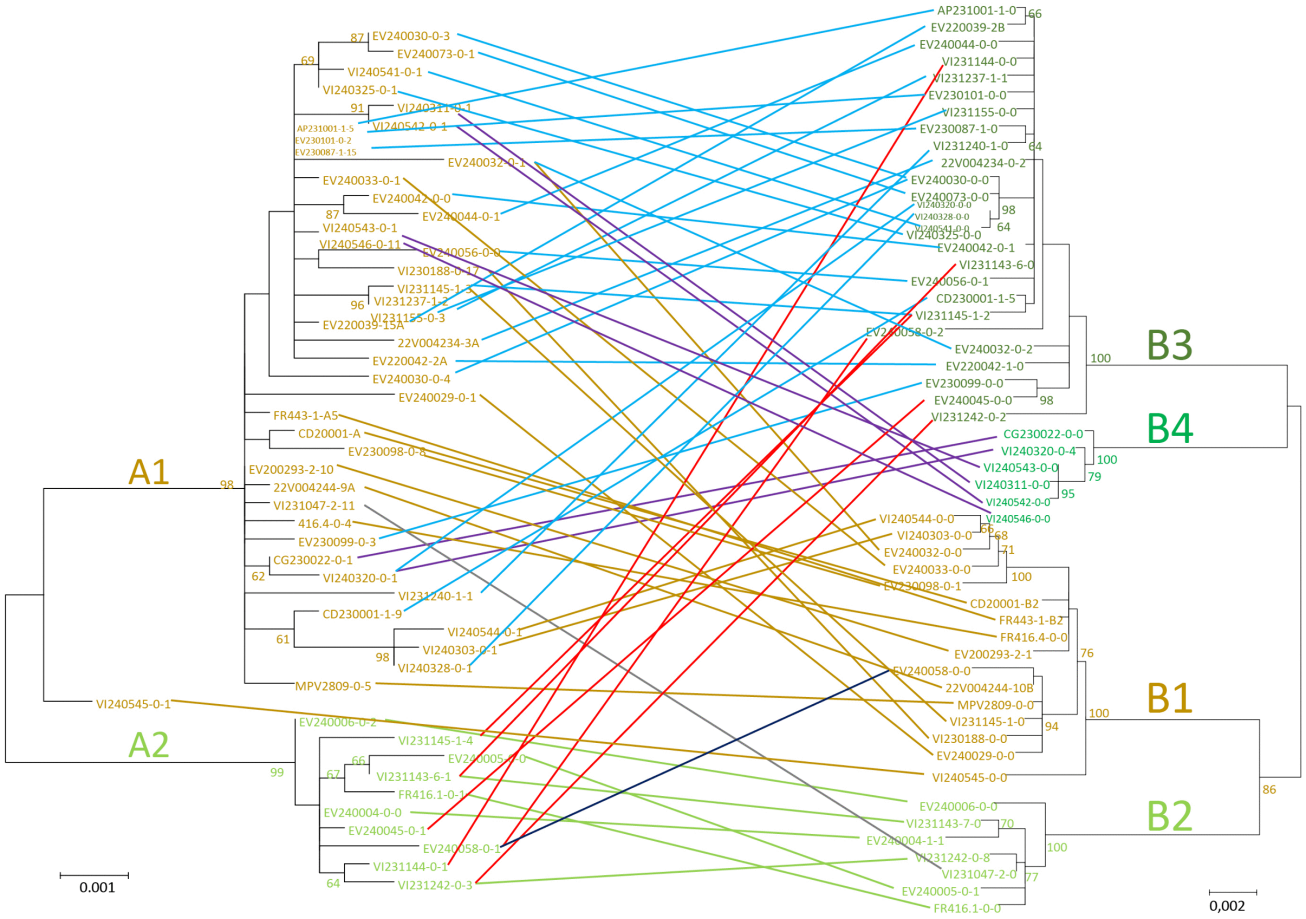
Maximum-likelihood phylogenetic tree of full-length DNA-A (left) and DNA-B (right) sequences from French ToLCNDV isolates collected between 2020 and 2024. Bootstrap values (n = 1000 bootstraps) are indicated at the corresponding nodes. Coloured lines indicate the associations between DNA-A and DNA-B component sequences for each isolate: yellow (A1 + B1), grey (A1 + B2), blue (A1 + B3), purple (A1 + B4), black (A2 + B1), green (A2 + B2), red (A2 + B3). For the six isolates presenting mixed infections, all viral components detected within the same isolate were considered to be associated.

Subgroups A1 and A2, as well as B1 and B2, were already present in 2020. Subgroup B3 was first detected in 2022, and B4 in 2023, indicating ongoing diversification of the DNA-B component. Multiple combinations of DNA-A and DNA-B subgroups were observed. The most frequent associations in plants infected with only one molecular subgroup for each DNA were A1 + B1, A1 + B3, A1 + B4 and A2 + B2, although additional combinations occurred sporadically (Fig 4). Among the six (or seven with VI231143 isolate) mixed infections identified in 2023–2024, B3 was detected in association with B1, B2 or B4, and with A1, A2 or both (S1 Table).

### 2.4. Time-structured phylogeographic analysis reveals the introduction timeline of ToLCNDV in southern France

The combination of a Bayesian skyline tree prior and an uncorrelated relaxed clock model provided the best fit for the Mediterranean ToLCNDV dataset for both DNA-A and DNA-B. The mean substitution rate was estimated at 3.8 x 10^-4^ substitutions/site/year for DNA-A (95% highest posterior density (HPD): 2.9 x 10^-4^ – 4.5 x 10^-4^) and 8 x 10^-4^ substitutions/site/year for DNA-B (95% HPD: 6.3 x 10^-4^ - 9.5 x 10^-4^).

**Fig 5:**
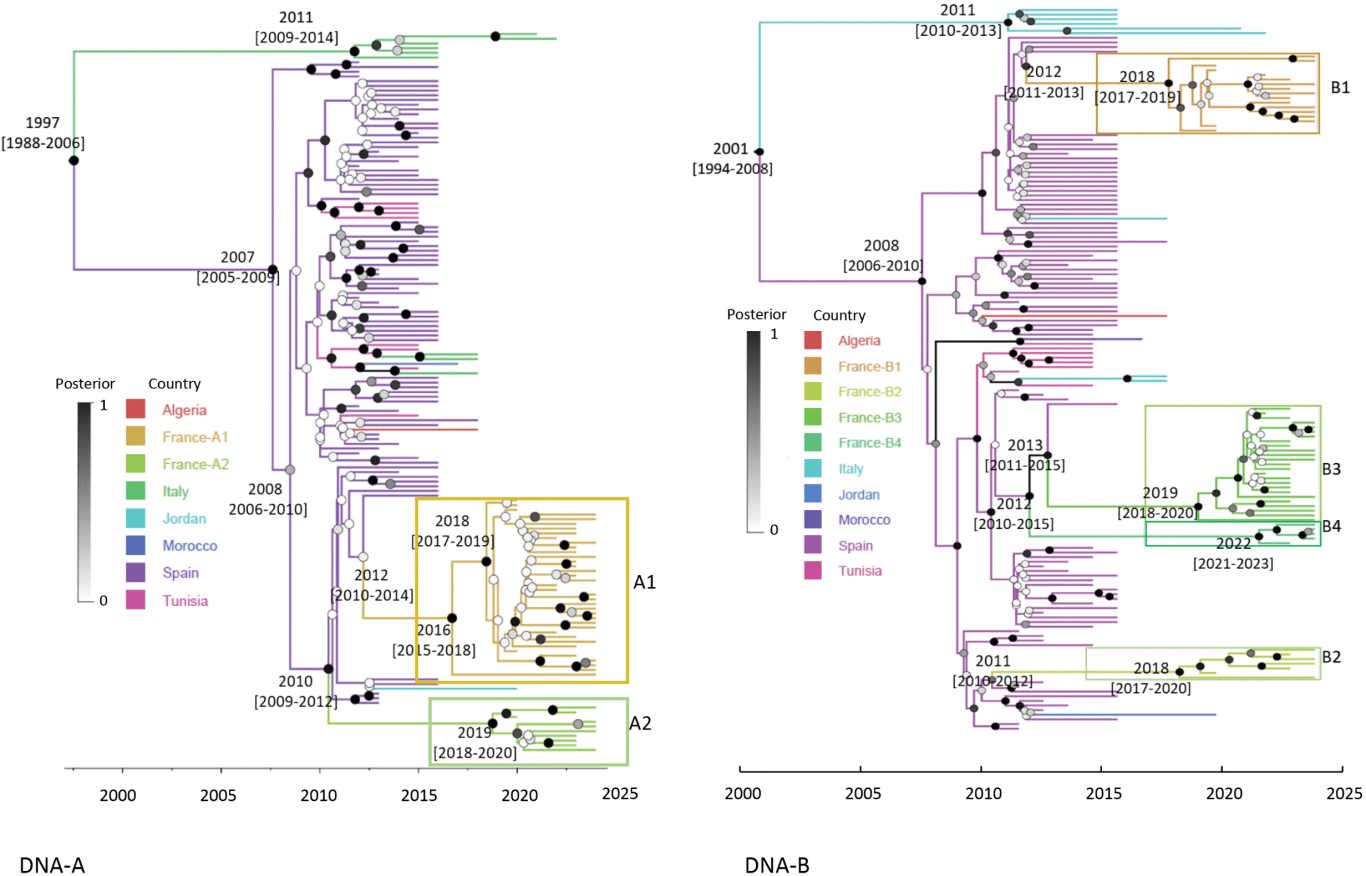
Time-scaled phylogenetic trees produced with BEAST for full-length DNA-A (left) and DNA-B (right) sequences of ToLCNDV-ES isolates collected in the Mediterranean Basin between 2012 and 2024. Analyses were performed using an uncorrelated relaxed molecular clock model with a Bayesian skyline tree prior. Nodes are shaded in grayscale according to posterior probability of clade support values. Branches are color-coded by geographic origin. French subgroups are highlighted with boxes. Estimated node ages and their associated 95% highest posterior density (HPD) intervals are indicated at the corresponding nodes.

Time-scaled maximum clade credibility trees for both the DNA-A and the DNA-B genomic component revealed two Mediterranean clades: a minor clade containing six isolates from southern Italy and a major clade comprising more than 100 isolates from across the Mediterranean region, including all French sequences (Fig 5). The divergence between these two clades was dated to 1997–2001 for both segments, with overlapping 95% HPD (1988–2006 for DNA-A and 1994–2008 for DNA-B), i.e., more than a decade before the first reported detection of ToLCNDV in the Mediterranean Basin.

Within the major Mediterranean clade, diversification was estimated to have begun around 2007–2008 (95% HPD 2006–2009 for DNA-A and 2006–2010 for DNA-B), whereas it was dated to 2011–2012 for the Italian minor clade (95% HPD 2009–2014 for DNA-A and 2010–2013 for DNA-B).

French isolates did not form a monophyletic group, supporting multiple introductions from the major Mediterranean clade. Divergence among French subgroups was dated to 2018–2019, for both DNA-A and DNA-B, except for subgroup B4, first detected in 2023, for which diversification was estimated at 2021–2022, albeit based on a limited number of samples. The ancestral nodes at the base of the French subgroups were dated between 2010 and 2013 (with overlapping 95% HPD intervals) close to the initial detection of ToLCNDV in the Mediterranean Basin (Spain, 2012), but long before its first detection in France.

Using a partial dataset that either completely excluded the Spanish isolates, or retained only 20 of them, had no impact on the clustering of the remaining isolates, and only a very limited effect on node dating (S3 Fig). The mean substitution rate remained essentially unchanged for DNA-A (4.1 x 10^-4^ substitutions/site/year, 95% HPD 2.9 x 10^-4^ – 4.5 x 10^-4^), but appeared lower for DNA-B when the Spanish isolates were completely excluded (6.9 x 10^-4^) or when only 20 Spanish isolates were retained (6.7 x 10^-4^ substitutions/site/year) compared with the estimate obtained using the full dataset. The corresponding 95% HPD intervals were 5.2 x 10^-4^ – 8.5 x 10^-4^ and 5.3 x 10^-4^ – 8.0 x 10^-4^, respectively. Using two different subsets of 20 Spanish isolates did not modify the result of the “partial” dataset. Nevertheless, the estimated substitution rate for DNA-B remained higher than that of DNA-A. Thus, the overrepresentation of Spanish isolates, including 53 isolates sampled in 2016, moderately affected the estimation of the substitution rate for DNA-B, but had little to no effect on that for DNA-A.

## 3. Discussion

This study applied a high-throughput Nanopore based circular DNA virus sequencing pipeline [24] to characterize ToLCNDV field isolates and investigate the evolution of Mediterranean ToLCNDV populations, since the first detection of the virus in France in 2020. Virus genome sequences reconstructed using the pipeline were fully consistent with Sanger data for the validated isolates, confirming the robustness and accuracy of the approach. The pipeline enabled reconstruction of full-length sequence variants for both ToLCNDV genomic components, providing direct insight into complex intra-host viral populations without the need for laborious subcloning, while reducing the risk of artefactual recombination associated with short read technologies [28,24].

Our results revealed substantial diversity among French ToLCNDV isolates, with two subgroups identified for DNA-A and four for DNA-B. The lack of monophyly among French subgroups supports multiple independent virus introductions into France [29]. Nevertheless, several subgroups were consistently detected between 2020 and 2024 in the same geographical areas, suggesting local persistence of the virus, possibly sustained by weed reservoirs. Indeed, ToLCNDV was detected in weed species growing around infected crops, including previously reported hosts [17], such as jimsonweed (*Datura*) and squirting cucumber (*Ecballium*) [17]. Viral populations characterized from weed samples clustered within the same subgroups as those infecting cultivated hosts. As a perennial cucurbit widely distributed across the Mediterranean Basin, *Ecballium* could represent a potential overwintering reservoir for ToLCNDV. However, ToLCNDV transmission by *Bemisia tabaci* between *Ecballium* and crops has been reported to be inefficient in Spain [30]. One potential bias of the study is that most sampling, except for weeds, was symptom-based. As a result, early infections and mildly symptomatic plants may have been overlooked, potentially leading to an underestimation of ToLCNDV prevalence and a biased view of its genetic diversity [31]. However, at least one systematic sampling was conducted independently of symptom expression. Both symptomatic and asymptomatic plants, the latter likely corresponding to recent infections, were sequenced, and no obvious differences were observed in the viral populations they contained.

Molecular analysis estimated divergence among French ToLCNDV subgroups between 2010 and 2013 for both DNA-A and DNA-B, i.e., at least six years prior to the first detection in France, and coinciding with ToLCNDV emergence in Spain. Diversification within the French subgroups was estimated at 2018–2019, likely corresponding to the timing of their introduction into France. Notably, subgroup B4 appears to have diverged more recently (2021–2022) and, although based on a limited sampling, we suggest that it may represent an additional, more recent introduction.

Available sequence data suggest a Spanish origin for the French isolates. However, this interpretation should be treated with caution, given the strong bias toward Spanish isolates detected between 2012 and 2016, which constitute the majority of publicly available ToLCNDV-ES genomic sequences (Fig 5; S3 Table). French isolates clearly differed from the minor Mediterranean clade containing isolates from southern Italy [32]. Notably, this Italian clade appears to have diverged in the early 2000s, more than a decade before the first detection of ToLCNDV in the Mediterranean Basin. This pattern suggests that isolates from Southern Italy have a distinct evolutionary trajectory and possibly an independent introduction.

For the major Mediterranean clade of ToLCNDV-ES, evolutionary analyses support a single introduction event around 2007–2008, with a convergent evolutionary history for DNA-A and DNA-B. The pathways by which ToLCNDV-ES was introduced into the Mediterranean Basin and subsequently into France remain unresolved. Seed transmission has been demonstrated in zucchini for the Italian subclade isolates [33], whereas Spanish isolates have been reported to be seedborne but not seed-transmitted in several cucurbit species [34,35].

Although DNA-A and DNA-B of ToLCNDV-ES appear to share a similar timing of introduction into the Mediterranean Basin, DNA-B exhibited greater genetic diversity with an estimated substitution rate approximately twice that of DNA-A (3.8 x 10^-4^ substitutions/site/year for DNA-A vs. 8 x 10^-4^ substitutions/site/year for DNA-B) even though the estimation for DNA-B was likely affected by the imbalanced dataset. These evolutionary rates fall within the range reported for other begomoviruses [36,37].

Recent estimates of ToLCNDV nucleotide substitution rates were approximately 7 x 10^‒4^ substitutions/nt/yr for DNA-A and 8.1 to 8.7 x 10^-4^ substitutions/nt/yr for DNA-B [38]. The higher substitution rate reported for DNA-A compared to our estimates may reflect the consideration of both Asian and Mediterranean ToLCNDV sequences in that study. Given that several ToLCNDV isolates and strains, including ToLCNDV-ES, have been described as intra- or interspecific recombinants for DNA-A [16], incorporation of these recombinant sequences may inflate substitution rate estimates. Notably, evolutionary rate estimates for DNA-B were consistent across studies.

For ToLCNDV-ES, the lower substitution rate observed for DNA-A relative to DNA-B may be explained by the presence of overlapping ORFs in DNA-A. Such genomic constraints increase the likelihood of non-synonymous mutations and are therefore subject to stronger purifying selection [39]. However, at the intra-host level, higher mutation frequencies in DNA-A than in DNA-B have been reported for another begomovirus [40], suggesting that selective and evolutionary dynamics may differ across biological scales.

In our study, overlapping regions among DNA-A ORFs were not explicitly accounted for in the estimation of dN/dS ratios. A single codon in the CP was identified as being under positive selection, with French isolates differing from most other ToLCNDV-ES sequences at this position. This pattern likely reflects, at least in part, the strong geographic structuring of genetic variation rather than selection alone, as begomovirus populations are often geographically structured [41], and recombination and demographic history can confound codon-level selection signals [36], but the fact that the S_147_ in the CP of French isolates is encoded by different codons AGC or TCA suggests a convergent evolution related, at least in part, to selection. The mutation also affects the putative AC5 protein in antisense orientation, resulting in a stop codon yielding a shorter protein for isolates presenting a TCA in the CP. Since all French isolates have the same S_147_ in their CP, the S_147_ CP mutation could explain potential functional or biological differences between the French isolates and the other ToLCNDV-ES, but differences among French isolates would probably be related to an impact of the mutation on AC5 and/or DNA structure rather than on the CP. Preliminary studies have not revealed any notable differences in host range or vector transmission between the French and Spanish ToLCNDV isolates tested so far. Further studies are needed to determine the possible functional or biological effects of the mutation, and whether they are mostly due to the changes in CP or in AC5. Therefore, its interpretation should be approached with caution given the constraints imposed by overlapping reading frames.

The high-throughput sequencing and analytical framework employed in this study enabled the characterization of mixed infections involving multiple DNA-A and/or DNA-B subgroups within individual plants, including both crops and weeds. Such complex intra-host virus populations may facilitate reassortment and recombination. Although several reassortment events were detected, it remains unclear whether these events occurred prior to or following introduction into France.

In contrast, recombination appeared rare among French ToLCNDV isolates. Only a single potential recombination event was detected in DNA-A, and its support was limited due to the low phylogenetic signal in the dataset. No clear recombination events were identified in DNA-B, despite sufficient intergroup diversity to allow their detection. Even in mixed-infected plants, long-read sequencing did not reveal low-frequency recombinant variants arising during virus replication.

This recombination inactivity contrasts with the reported recombination-prone nature of begomoviruses [42,43]. One possible explanation is that co-infections in France are relatively recent, limiting the time available for recombinant forms to emerge and establish. In perennial weed hosts such as *Ecballium,* where viruses may likely persist for multiple years, long-term viral co-infections may lead to the emergence of recombinants with enhanced fitness, as reported for other begomoviruses [44]. It is likely that recombination detection was influenced by methodological limitations, such as the number and geographic distribution of the sampled fields, rather than reflecting a true biological absence of recombination.

The multiyear persistence of distinct molecular subgroups of ToLCNDV-ES in France suggests that the virus is now established in the region, potentially facilitated by its maintenance in perennial weed reservoirs. A deeper understanding of the long-term evolutionary dynamics of ToLCNDV-ES in weed reservoirs and cultivated hosts, its potential to generate novel recombinants, and the relative fitness of its subgroups, alone or in mixed infections, will be critical for preventing further geographic expansion of the virus.

## 4. Materials and methods

### 4.1. Plant samples collection

French ToLCNDV isolates were obtained through field sampling in collaboration with private breeders, farm advisers, agricultural extension services, and official quarantine authorities. Samples were collected between 2020 and 2024 from cultivated plants, including zucchini, melon, and cucumber, as well as from weed species such as *Datura stramonium* (jimsonweed) and *Ecballium elaterium* (squirting cucumber)*,* across the French départements (counties) of Gard, Bouches-du-Rhône, and Vaucluse (Fig 1; S1 Table). Collection was based on symptomatic plants, complemented by random sampling of asymptomatic crops and weed species located in the vicinity of infecting fields. In 2024, a systematic sampling of 30 zucchini plants was conducted within a field trial independently of symptom expression. The samples were tested by ELISA for the presence of ToLCNDV and other viruses, and five ToLCNDV-positive samples were subsequently selected for ONT sequencing (isolates VI240303-VI240328; S1 Table).

### 4.2. DNA extraction and ONT sequencing

The presence of ToLCNDV in the collected samples was confirmed by DAS-ELISA with a commercial ToLCNDV antiserum (Agdia EMEA, Soisy sur Seine, FR), following the manufacturer’s recommendations.

Total DNA was extracted as previously described [45]. Isolated DNA was amplified and submitted to long read ONT sequencing following the established protocol [28,24]. Briefly, extracted total DNA was submitted to circular DNA enrichment via RCA, debranched using NEBNext FFPE DNA Repair and NEBNext Ultra II And Repair/dA-Tailing modules. DNA clean-up was subsequently performed using AMPure XP Beads (Beckman Coulter, USA) before ligating adapters with the Nanopore Ligation Sequencing Kit v14, following the manufacturer’s protocol. Next, the DNA was used to prepare libraries for ONT long read sequencing. Ten to twelve samples, without negative controls, were barcoded using the Oxford Nanopore Native Barcoding kit v14, pooled, adapter ligated with the Nanopore Ligation Sequencing Kit v14 and loaded into a Nanopore flow-cell (R10.4.1). Sequencing runs were performed for 48-72h. Raw sequencing data were basecalled using Dorado (model: dna_r10.4.1_e8.2_400bps_sup@v5.2.0) (https://github.com/nanoporetech/dorado), followed by demultiplexing and trimming using Porechop (https://github.com/rrwick/Porechop). Processed data were uploaded to the Genome Detective platform [25] for virus variant analysis, providing for each sample a list of haplotypes represented by at least 10 reads. Variants supported by fewer than ten sequences were excluded from the analysis, as indels occur frequently in homopolymeric regions in these sequences. Minor haplotype sequences supported by at least ten sequences were manually inspected for obvious sequencing errors in homopolymeric regions, which were corrected when identified.

### 4.3. Virus cloning and Sanger sequencing

Four isolates from 2020 and four isolates from 2022 (S1 Table) were cloned using RCA products digested with *Bam*HI and ligated into a *Bam*HI-linearized pBluescript (KS)+ vector (Strategene, La Jolla, CA). After transformation of *Escherichia coli* (strain DH5α), the cloned viral DNAs were amplified with universal primers (M13-21 and M13rev) and submitted for sanger sequencing (GenoScreen, France) with M13-21, M13rev, and internal ToLCNDV-A-1000F and ToLCNDV-B-1700-F primers [8; S4 Table]. The chromatograms were analysed using Chromas (https://technelysium.com.au/wp/chromas/), and viral full-length sequences were reconstructed. Eight samples collected in 2023 were partially amplified with virus-specific primers (S4 Table) and subsequently sequenced (GenoScreen, France). These sequences were compared with those obtained by ONT long-read sequencing.

### 4.4. Full-length sequence dataset and Multiple Sequence Alignment workflow

Complete sequences of 50 French ToLCNDV isolates collected between 2020 and 2024 (S1 Table), together with 110 Mediterranean ToLCNDV isolates retrieved from GenBank (S3 Table), were included in the analyses. 48 French isolates, as well the Spanish reference isolate ES13–35, were sequenced using ONT long-read sequencing.

In addition to 48 isolates sequenced by ONT, two French isolates (22V0044 and CD20001) were included in the analyses. Isolate 22V0044 was sequenced by Sanger sequencing only, while isolate CD20001, corresponding to the first ToLCNDV isolate detected in France [8] had previously been sequenced by both Sanger sequencing (accessions MW310624-MW310625 for DNA-A and DNA-B, respectively) and Illumina short read sequencing.

Hereafter, the datasets comprising 50 and 160 isolates will be referred to as the “French” and “complete” datasets, respectively.

For French isolates with mixed infections including different molecular subgroups, the major haplotype representing each subgroup was retained for analysis. Separate analyses such as genetic selection and recombination were performed using both the “complete” and “French” datasets.

For large datasets, full-length DNA-A (2,740 nt) and DNA-B (2,684 nt) sequences were aligned using MAFFT (Multiple Alignment using Fast Fourier Transform algorithm) with default parameters implemented in Job Dispatcher platform (https://ebi.ac.uk/jdispatcher/) [46]. Smaller datasets were aligned using the MUSCLE algorithm [47].

### 4.5. Recombination and selection analysis

Preliminary screening for recombination events was done on the complete (160 sequences) and French (50 isolates, yielding 48 different sequences for DNA-A and 51 different sequences for DNA-B) datasets using the Genetic Algorithm for Recombination Detection (GARD), implemented on the Datamonkey server (https://www.datamonkey.org/). The same datasets were subsequently analysed with RDP4 [48], using seven independent detection methods: RDP, GENECONV, Bootscan, Siscan, 3Seq, Chimaera, and Maxchi, with p < 0.01 significance threshold. Recombination breakpoints identified by GARD and confirmed by at least three independent RDP4 analyses were retained. Potential recombinations among ToLCNDV sequences were independently assessed using SplitsTree (version 6.5.1) [49] to visualize phylogenetic conflicts [50]. The significance of recombination signals was validated using the Phi test [51].

Seven open reading frames (ORFs) for DNA-A and two for DNA-B were obtained from all ToLCNDV sequences and validated using the NCBI ORFfinder (https://www.ncbi.nlm.nih.gov/orffinder/). The coding sequences were aligned with the MUSCLE algorithm implemented in MEGA 7 [52] and subsequently analysed for signatures of positive selection, defined by a higher rate of nonsynonymous (dN) than synonymous (dS) substitutions, using the HyPhy-based DataMonkey platform (https://www.datamonkey.org/). Episodic diversifying selection was assessed with MEME (Mixed Effects Model of Evolution) [53]. Pervasive selection was evaluated using FUBAR (Fast, Unconstrained Bayesian AppRoximation) [54]. The thresholds were set at p < 0.01 for MEME and posterior probability > 95% for FUBAR.

### 4.6. Phylogenetic analysis

Maximum Likelihood phylogenetic trees were constructed with MEGA 7 after selecting the appropriate substitution model [52] for the aligned sequences. For DNA-A sequences, the Tamura 3-parameter model with a discrete Gamma distribution (T92 + G) and 1000 bootstrap replicates was applied. For DNA-B sequences, the same model was used with the additional assumption of a proportion of invariant sites (T92 + G + I).

### 4.7. Time-structured phylogeographic analysis

Phylogeographic analysis was conducted using the BEAST v1.10.4 software package to infer the spatiotemporal dynamics of viral spread [55]. Full-length ToLCNDV genomic sequences were aligned with MAFFT and subsequently processed with BEAUTi [56], with sampling dates and geographic origins incorporated as discrete traits. The HKY nucleotide substitution model was selected with estimated base frequencies, and a gamma distribution (4 categories) was applied. The temporal signal was evaluated with TempEst [57]. Two molecular clock parameters (strict molecular clock vs. uncorrelated relaxed clock) and three tree priors (coalescent constant size, exponential growth and Bayesian skyline) were tested. MCMC chains were run for 10 million iterations, and outputs were assessed with Tracer to ensure proper convergence. If the convergence was not obtained after 10M runs, the analysis was performed again with 20M runs, and 100M runs for DNA-B. After discarding 10% burn-in, a maximum clade credibility (MCC) tree was generated using TreeAnnotator and then visualized using FigTree v1.4.4 (https://tree.bio.ed.ac.uk/software/figtree/).

To assess the impact of the strong overrepresentation of Spanish isolates in the dataset, BEAST analyses were performed on two partial DNA-A and DNA-B datasets: (1) dataset completely excluding sequences from Spain 2012–2016, and (2) a dataset retaining 20 Spanish sequences, limited to 3–4 randomly selected sequences per year (2012–2016). In both cases, analyses were conducted using an uncorrelated relaxed molecular clock, a Bayesian skyline prior, and 10 million MCMC iterations.

## Supporting information

S1 Table French ToLCNDV isolates sequenced in the study, indicating their major haplotypes and corresponding molecular subgroups. Abbreviations: B. du Rhône, Bouches-du-Rhône; St Remy de P, Saint-Rémy-de-Provence.
(XLSX)

S2 Table Comparison of Sanger and Nanopore sequencing results for French ToLCNDV isolates. (XLSX)

S3 Table Mediterranean ToLCNDV isolates (excluding French isolates) used for comparative and phylogenetic analyses in the study. (XLSX)

S4 Table Primers used for amplification and sequencing of ToLCNDV isolates. (XLSX)

S1 Fig Relative frequencies (%) of the major haplotypes obtained for DNA-A (top) and DNA-B (middle) in 48 French ToLCNDV isolates after ONT sequencing. For each sample, the four most abundant haplotypes are displayed according to their relative frequencies, while all remaining minor haplotypes are grouped in white. Histograms outlined in red correspond to haplotypes belonging to a molecular subgroup different from the major haplotype detected in the sample. Asterisks above the histograms indicate samples considered to show strong evidence (asterisks without brackets) or limited evidence (asterisks in bracket) of mixed infection involving different molecular subgroups. Samples collected from weeds are indicated by a triangle for *Datura stramonium* and a square for *Ecballium elaterium*. Bottom: chromatogram obtained after Sanger sequencing of DNA-B of isolate VI231242 with primer ToLCNDV-B-1700-F, showing double peaks corresponding to a mixed infection with molecular subgroups B2 and B3, and the double sequence after position 510 (2235 in the genome) due to the deletion of one “G” in sequences from subgroup B3.
(TIF)

S2 Fig Split decomposition network produced with SplitsTree for DNA-A (top) and DNA-B (bottom) sequences of French ToLCNDV isolates. **Molecular subgroups (A1-A2 for DNA-A; B1-B4 for DNA-B) are indicated.** The boxed alignment shows parsimony-informative sites distinguishing DNA-A sequence VI240545-0-1 from isolates belonging to subgroups A1 (brown) and A2 (green). The positions of nucleotide substitutions within the DNA-A genome are indicated above the alignment. Dots denote nucleotides identical to VI240545-0-1.
(TIF)

S3 Fig Time-scaled phylogenetic trees produced with BEAST for full-length DNA-A (top) and DNA-B (bottom) sequences of ToLCNDV-ES isolates collected in the Mediterranean Basin between 2012 and 2024. Left: dataset excluding the Spanish isolates from 2012-2016. Right: dataset including 20 Spanish isolates (3–4 per year each between 2012 and 2016). Analyses were performed using an uncorrelated relaxed molecular clock model with a Bayesian skyline tree prior. The viral genome sequences used in this study have been deposited in the NCBI GenBank database under accession numbers PX842845-PX842948 (S1 Table). Oxford Nanopore sequencing data generated in this study have been deposited in the European Nucleotide Archive (ENA) under study accession **PRJEB114816**.
(TIF)
